# Factors associated with pretreatment and treatment dropouts: comparisons between Aboriginal and non-Aboriginal clients admitted to medical withdrawal management

**DOI:** 10.1186/1477-7517-10-38

**Published:** 2013-12-10

**Authors:** Xin Li, Huiying Sun, David C Marsh, Aslam H Anis

**Affiliations:** 1 Antai College of Economics & Management, Shanghai Jiao Tong University, 535 Fhuazhen Rd, Shanghai, China; 2 Centre for Health Evaluation and Outcome Sciences, 588-1081 Burrard Street, Vancouver, B.C. V6Z 1Y6, Canada; 3 Northern Ontario School of Medicine, Vancouver, B.C. V6Z 1Y6, Canada; 4 School of Population and Public Health, University of British Columbia, Vancouver, B.C. V6Z 1Y6, Canada

**Keywords:** Aboriginal, Housing, Pretreatment dropout rate, Treatment dropout rate, Withdrawal management, Substance use disorders, Detoxification

## Abstract

**Background:**

Addiction treatment faces high pretreatment and treatment dropout rates, especially among Aboriginals. In this study we examined characteristic differences between Aboriginal and non-Aboriginal clients accessing an inpatient medical withdrawal management program, and identified risk factors associated with the probabilities of pretreatment and treatment dropouts, respectively.

**Methods:**

2231 unique clients (Aboriginal = 451; 20%) referred to Vancouver Detox over a two-year period were assessed. For both Aboriginal and non-Aboriginal groups, multivariate logistic regression analyses were conducted with pretreatment dropout and treatment dropout as dependent variables, respectively.

**Results:**

Aboriginal clients had higher pretreatment and treatment dropout rates compared to non-Aboriginal clients (41.0% vs. 32.7% and 25.9% vs. 20.0%, respectively). For Aboriginal people, no fixed address (NFA) was the only predictor of pretreatment dropout. For treatment dropout, significant predictors were: being female, having HCV infection, and being discharged on welfare check issue days or weekends. For non-Aboriginal clients, being male, NFA, alcohol as a preferred substance, and being on methadone maintenance treatment (MMT) at referral were associated with pretreatment dropout. Significant risk factors for treatment dropout were: being younger, having a preferred substance other than alcohol, having opiates as a preferred substance, and being discharged on weekends.

**Conclusions:**

Our results highlight the importance of social factors for the Aboriginal population compared to substance-specific factors for the non-Aboriginal population. These findings should help clinicians and decision-makers to recognize the importance of social supports especially housing and initiate appropriate services to improve treatment intake and subsequent retention, physical and mental health outcomes and the cost-effectiveness of treatment.

## Background

Substance use disorders (SUD) are a common problem and a major issue of concern for Canada’s Aboriginal population [1]. Aboriginal people are also overrepresented among HIV and AIDS cases in Canada. They represented 3.8% of the Canadian population [2], and yet accounted for 8.0% of all prevalent HIV infections and 12.5% of new infections in 2008. The estimated new infection rate among Aboriginal people was about 3.6 times higher than that among non- Aboriginal people. In addition, the proportion of estimated new HIV infections in 2008 among Aboriginal people who inject drugs (66%) was also much higher than that among all Canadians (17%) [3].

British Columbia (BC) had the second largest Aboriginal population among all Canadian provinces, representing 5% of the provincial population, and the census metropolitan area of Vancouver had the largest Aboriginal population of any city in BC [2]. Vancouver’s Downtown Eastside (DTES) is the most impoverished urban neighborhood in Canada and is the centre of the injection drug epidemic in Vancouver; 4,700 injection drug users (IDUs) were estimated to live in DTES in 2000 [4]. Previous studies on Vancouver have shown that Aboriginal people are overrepresented among injection drug users (IDUs) in DTES, are becoming HIV positive at twice the rate of non-Aboriginal IDUs, have a six-fold higher incidence of acute hepatitis C infection (HCV) and are more likely to be co-infected with HIV and HCV compared to the non-Aboriginal population. In addition, they also have higher rates of drug-induced deaths [5].

Although previous studies have shown that addiction treatment programs are effective in reducing substance use, in improving clients’ health and social function and in reducing public health and safety risks [6-8], they face high pretreatment and treatment dropout rates, ranging from 28% to 50% [9-11] and 20% to 46% [12-14], respectively. Several studies have examined correlates of treatment dropout rates from drug and alcohol detoxification programs [15-22]. Although there was no consensus on whether or not specific features of a detoxification program significantly improve client retention, these studies did identify several client-related factors that were related to the treatment dropout rate, such as age [15,16,21], education [16,18], living condition [18], presence of legal problems [16,17], substance use pattern [15,17,19,21], and timing of welfare payments [21]. However, little research has been carried out to provide clinically relevant research on appropriate treatment for Aboriginal populations. The only study, which examined risk factors associated with dropout among Aboriginal people, found that a preferred drug other than alcohol and self-referral were two significant predictors of treatment dropout for this group of people [20].

Vancouver has a wide range of addiction services including a multilevel withdrawal management continuum [23]. The aim of this study was to examine characteristic differences between Aboriginal and non-Aboriginal clients accessing an inpatient medical withdrawal management program in Vancouver. In addition, we identified risk factors associated with pretreatment and treatment dropout rates for the two populations. Specifically, based on previous literature, the present study hypothesized that in addition to the factors such as socio-demographics, current substance use, and IDU-related diagnoses, day of discharge in relation to treatment dropout might also affect treatment dropout as it is possible that different staffing on weekdays versus weekends or some special events, such as once-a-month welfare check issuance (welfare Wednesday), might contribute to client dropout.

## Methods

### Study sample

The sample consisted of 2231 individuals who called ACCESS 1 requesting service at Vancouver Detox (VD) between July 1, 2003 and June 30, 2005. For those clients who called multiple times, only the first referral during the study period was included. Despite the multicultural nature of Vancouver, no ethnic group other than European/White and Aboriginal represented 5% or more of the total in our sample, therefore, the present study focused on the Aboriginal versus non-Aboriginal groups. Ethical Approval was obtained from the Behavioural Research Ethics Board at the University of British Columbia.

### Treatment setting

VD offers a medically managed 24-bed mixed-gender inpatient withdrawal management treatment in Vancouver, British Columbia. It is staffed by a multidisciplinary team, consisting of addiction medicine physicians, nurses, health care workers, intake workers, and alcohol and drug (A&D) counselors. It provides 24-hour nursing staff, onsite medical assessment and treatment, and medical management of withdrawal symptoms and other identified health concerns. Vancouver Detox can be accessed free of charge by anyone registered with a provincial medical services plan and living in the Vancouver Coastal Health catchment area. Thus, there are no financial barriers to treatment.

The entry point to VD is by ACCESS 1, a central telephone intake service. After an initial telephone screening, ACCESS 1 determines whether the client is eligible for the service. The main eligibility criteria for VD are: 1) above the age of fifteen, 2) having unstable medical or psychiatric conditions, and/or 3) having a history predictive of severe withdrawal, and 4) not pregnant at the time of call. If the client meets the criteria for VD, he/she is either admitted to VD immediately or placed on a waitlist for a bed to become available. Once admitted, the clinical staff complete a comprehensive assessment and develop an individualized treatment plan. During the admission, the client participates in various rehabilitative programs, including such activities as: individual counseling, educational groups, 12-step programs, acupuncture and other alternate therapies. Clients admitted on methadone maintenance are maintained on this medication throughout the admission. Clients are either discharged by successfully completing the program or voluntarily dropping out by terminating against medical advice (AMA). Upon discharge clients are referred to other services as required.

### Data

During the initial phone interview, ACCESS1 staff collect some basic information such as age, gender, address, and conduct a brief addiction assessment using a Vancouver Coastal Health Authority on-line, real-time system, Primary Access Regional Information System (PARIS) database. Once a client is admitted to the VD, an intake worker verifies the information inputted by ACCESS1 and completes a more comprehensive assessment which is entered into the database. During the admission, the VD staff update the clinical information in PARIS until the client is discharged.

The PARIS database is comprehensive and includes many variables. For the purpose of this study the following variables were extracted: clients’ unique identification (PARIS number), demographic information (age, gender, ethnicity, parenting status), housing information (no fixed address (NFA) vs. others), system characteristics (referral, admission, and discharge dates), substance use disorder-related information (substances recently used, primary preferred substance(s), discharge reason, if on existing methadone maintenance, and pre-existing IDU-related diagnoses such as hepatitis C (HCV) or HIV infection).

### Statistical analyses

Contingency table analysis (the Chi-square test) and the Wilcoxon rank-sum test were used for bivariate comparisons of categorical and continuous variables, respectively. For both Aboriginal and non-Aboriginal groups, logistic regression analyses were performed separately with pretreatment dropout and treatment dropout as the dependent variables. For comparison purpose, variables that were significant at *p* < 0.25 in either bivariate analysis were subsequently entered into the multivariate logistic regression models for both Aboriginal and non-Aboriginal groups. Odds ratios (ORs) and 95% confidence intervals (CIs) were computed. All statistical analyses were performed using SAS 8.2 software program [24].

## Results

Among 2231 unique clients who were referred by ACCESS1 to VD during the study period, 451 (20%) clients self identified as Aboriginal. Compared with the non-Aboriginal group at referral, the Aboriginal group was younger, more likely to be female and NFA, and had higher rates of HIV and HCV. In addition, the Aboriginal group had proportionately more poly-drug users (Table 1).

**Table 1:** Comparisons between aboriginal and non-aboriginal clients who were referred to Vancouver Detox (N = 2231)

**Variable**	**N**	**Aboriginal N (%)**	**Non-aboriginal N (%)**	**P value**
	2231	451	1780	.
Age (years (SD))		37.8 (9.1)	41.2 (11.4)	<0.0001
Female	788	207 (46.0)	581 (32.8)	<0.0001
Dependent children	184	40 (8.9)	144 (8.1)	0.5910
Self-referrals	2126	434 (96.2)	1692 (95.1)	0.2928
No fixed address (NFA)	362	105 (23.3)	257 (14.4)	<0.0001
HIV positive	153	71 (15.7)	82 (4.6)	<0.0001
Hepatitis C	671	207 (45.9)	464 (26.1)	<0.0001
Alcohol as primary substance of choice	1321	286 (64.6)	1035 (59.1)	0.0363
Cocaine as primary substance of choice*	759	200 (45.1)	559 (31.9)	<0.0001
Opiates as primary substance of choice**	381	72 (16.3)	309 (17.7)	0.4889
Poly-drug use	661	160 (36.2)	501 (28.6)	0.0019
Methadone prescribed	92	20 (4.4)	72 (4.0)	0.7101

*Cocaine group includes cocaine, crack cocaine.

**opiates group includes heroin, methadone, oxycodone, other opiates.

Among the referred clients, 767 (34%) dropped out without engaging in treatment. Among these, 185 clients were Aboriginal. The pretreatment dropout rate for Aboriginals was therefore 41%, whereas the rate was 32.7% for the non-Aboriginal group. For 1464 clients who initiated treatment, 309 (21.1%) left the service AMA. Among these, 69 were Aboriginal. As a result, the treatment dropout rates for the Aboriginal and non-Aboriginal groups were 25.9% and 20.0%, respectively. In addition, our data indicated that the pretreatment dropouts accounted for the major proportion of total dropouts for both groups, representing 72.8% for the Aboriginal group and 70.8% for the non-Aboriginal group.

Results of the bivariate and multivariate analyses on pretreatment dropout for both the Aboriginal and non-Aboriginal groups are presented in Table 2. For the Aboriginal group, without fixed address (OR = 1.75, 95% CI 1.10 – 2.79) was the only significant predictor of pretreatment dropout. For the non-Aboriginal group, our results showed that females (OR = 0.72, 95% CI 0.57 – 0.91) were less likely to be pretreatment dropouts than their counterparts. On the other hand, the probability of pretreatment dropout was higher for people without fixed address (OR 1.87, 95% CI 1.40 – 2.48). Clients whose primary preferred substance was alcohol were more likely to be pretreatment dropouts than clients whose preferred substance was other than alcohol (OR = 1.77, 95% CI 1.36 – 2.30). In addition, clients on methadone maintenance treatment (MMT) at referral had higher probability of pretreatment dropout (OR = 2.30, 95% CI 1.40 – 3.79).

**Table 2:** Bivariate and multivariate logistic regression of pretreatment dropout for aboriginal and non-aboriginal clients

	**Aboriginal (N = 451)**			**Non-aboriginal (N = 1780)**		
	**Bivariate analysis**	Multivariate analysis*		**Bivariate analysis**	Multivariate analysis**	
**Variables**	**P value**	**OR**	**95% CI**	**P value**	**OR**	**95% CI**
Age	0.48	0.99	(0.96, 1.01)	0.16	0.99	(0.98, 1.00)
Female	0.24	0.97	(0.64, 1.48)	0.00	0.72^†^	(0.57, 0.91)
Dependent children (Yes)	0.14	0.56	(0.27, 1.19)	0.04	0.73	(0.49, 1.09)
No fixed address (NFA)	0.01	1.75^†^	(1.10, 2.79)	0.00	1.87^†^	(1.40, 2.48)
Alcohol as primary substance of choice	0.01	1.40	(0.86, 2.25)	0.00	1.77^†^	(1.36, 2.30)
Cocaine as primary substance of choice	0.02	0.77	(0.49, 1.19)	0.23	1.25	(0.97, 1.61)
Opiates as primary substance of choice	0.23	0.93	(0.49, 1.77)	0.38	0.99	(0.72, 1.37)
Poly-drug use (Yes)	0.03	0.71	(0.43, 1.17)	0.73	0.99	(0.74, 1.32)
Methadone prescribed (Yes)	0.58	1.04	(0.38, 2.83)	0.01	2.30^†^	(1.40, 3.79)

*P-value for Hosmer and Lemeshow goodness of fit test was 0.63.

**P-value for Hosmer and Lemeshow goodness of fit test was 0.53.

†Statistically significant at p < 0.05.

Table 3 presents the bivariate and multivariate analyses on treatment dropout for both the Aboriginal and non-Aboriginal groups. Our final multivariate logistic analysis showed that for the Aboriginal group, females (OR = 2.07, 95% CI 1.03 – 4.15) and clients who reported to have HCV (OR = 4.91, 95% CI 2.43 – 9.94) were more likely to drop out of treatment. In addition, compared to clients who discharged in other days, clients who discharged during the weekend (OR = 2.28, 95% CI 1.04 – 5.01) or during 3-day welfare check issue periods (OR = 2.84, 95% CI 1.06 – 7.59) were more likely to be treatment dropouts. In the non-Aboriginal group, older clients (annual change OR = 0.97, 95% CI 0.96 – 0.99) and clients whose primary preferred substance was alcohol (OR 0.72, 95% CI 0.53 – 0.99) were less likely to drop out of treatment. On the other hand, clients who used opiates as primary preferred substance (OR = 1.63, 95% CI 1.08 – 2.46), and clients who discharged during weekend (OR = 2.44, 95% CI 1.71 – 3.46) were more likely to be treatment dropouts. There was no significant impact of welfare issuance on non-Aboriginals.

**Table 3:** Bivariate and multivariate logistic regression of treatment dropout for aboriginal and non-aboriginal clients

	**Aboriginal (N = 266)**			**Non-aboriginal (N = 1198)**		
	**Bivariate analysis**	Multivariate analysis*		**Bivariate analysis**	Multivariate analysis**	
**Variables**	**P value**	**OR**	**95% CI**	**P value**	**OR**	**95% CI**
Age	0.22	1.00	(0.96, 1.04)	0.00	0.97^†^	(0.96, 0.99)
Female	0.02	2.07^†^	(1.03, 4.15)	0.18	1.00	(0.72, 1.37)
Dependent children (Yes)	0.09	2.15	(0.83, 5.59)	0.93	1.07	(0.64, 1.80)
No fixed address (NFA)	0.32	1.53	(0.71, 3.31)	0.11	1.09	(0.70, 1.70)
Hepatitis C (Yes)	0.00	4.91^†^	(2.43, 9.94)	0.02	1.41	(0.98, 2.02)
HIV positive (Yes)	0.48	0.49	(0.20, 1.17)	0.22	0.47	(0.21, 1.07)
Alcohol as primary substance of choice	0.04	1.21	(0.59, 2.48)	0.00	0.59^†^	(0.40, 0.85)
Cocaine as primary substance of choice	0.02	1.62	(0.77, 3.40)	0.81	0.73	(0.51, 1.05)
Opiates as primary substance of choice	0.00	2.09	(0.89, 5.05)	0.00	1.63^†^	(1.08, 2.46)
Poly-drug use (Yes)	0.01	1.28	(0.59, 2.78)	0.01	0.93	(0.63, 1.39)
Days of discharge^‡^	0.22			0.00		
Weekend		2.28^†^	(1.04, 5.01)		2.44^†^	(1.71, 3.46)
Welfare check issue period		2.84^†^	(1.06, 7.59)		1.51	(0.96, 2.38)

*P-value for Hosmer and Lemeshow goodness of fit test was 0.60. **P-value for Hosmer and Lemeshow goodness of fit test was 0.84.

†Statistically significant at p < 0.05.

^‡^The reference category was being discharged on other weekdays. Welfare check issue periods were defined as welfare Wednesday, one day before (Tuesday) and one day after (Thursday).

## Discussion

Aboriginal people in Canada are facing a lot of social challenges. Historical policies that legalized racial identities and systemic inequity, and cast Aboriginal people to the margins of Canadian societies still extract considerable influence on the social, cultural, political and spiritual inequities that Aboriginal people endure [25]. Specifically, Aboriginal adults are more likely to experience social exclusion, less likely to access economic resources and opportunities such as participation in paid work and therefore more likely to have low income and be homeless [25] and are overrepresented in custody and community correctional programs [26]. In addition, Aboriginal children are overrepresented in the Canada child welfare system [27]. Moreover, they have been shown to bear a disproportionate burden of illness, substance use disorders, and disease in Canadian society [1]. In our sample, Aboriginal individuals, who accounted for approximately 5% of the BC provincial population, comprised 20% of clients in the VD. In addition, our findings showed that Aboriginal clients appeared to be different from their non-Aboriginal counterparts in terms of age, gender, housing status, substance use pattern and pre-existing IDU-related diagnoses. The multivariate logistic analyses also revealed different risk factors of pretreatment and treatment dropout rates for the two groups. These findings reinforce the need for culturally appropriate services for Aboriginals who are at a greater risk for negative outcomes within the current addiction treatment system.

It was interesting to show that rather than demographic or substance use-related information, lack of a fixed address was the only factor that was significantly associated with pretreatment dropout for Aboriginal clients. To prevent attrition early in the treatment phase for this group of clients, our findings suggest that interventions such as providing these homeless clients with temporary accommodations will allow treatment staff to more easily reach them once beds are available and to cater to their immediate health needs. This could also then improve treatment initiation significantly. Our findings are consistent with the findings found by “At Home” study. In 2008, the Government of Canada allocated $110 million to the Mental Health Commission of Canada (MHCC) to undertake the “At Home” study, a four-year project in five cities that aimed to provide practical, meaningful support to Canadians experiencing homelessness and mental health problems. The preliminary result of the study has shown that implementing a Housing First strategy can significantly improve homeless clients with mental illness and/or SUD to access the type of care that is vital for recovery [28].

For the non-Aboriginal group, in addition to NFA, we found that several other factors such as being male, alcohol as a preferred substance, and being on MMT at referral were also associated with dropout before treatment. Previous studies have shown that longer delay between the initial phone contact and the scheduled appointment is negatively associated with treatment initiation [29,30]. However, the tolerance for delay and reasons for delay might vary for different clients. Specifically, those with alcohol alone, as the problem substance, may be receiving treatment services from a primary care physician or emergency room and be prescribed benzodiazepines for outpatient management of withdrawal and thus preventing the need for admission. On the other hand, clients enrolled in MMT at referral might have a longer wait time than other clients because of the need to coordinate the date and time of last dose taken in the community and to maintain continuity of MMT.

With regards to treatment dropout, our results also showed completely different sets of factors that were associated with treatment dropout AMA for the Aboriginal and non-Aboriginal groups. Specifically, Aboriginal but not other clients being HCV positive was associated with increased risk of leaving AMA compared to those who did not have this disease. HCV + status may be a marker for more severe dependence and injection drug use [31]as well as increased social marginalization [32,33]. In addition, previous literature has shown that there are significant increases in hospital admission, emergency department admission, emergency calls and deaths shortly after the distribution of monthly welfare cheques [34,35]. Our data showed that this was also a factor for Aboriginal clients at VD.

In contrast to our findings, Callaghan’s study found that a preferred drug other than alcohol and self-referral were significantly associated with treatment dropout for Aboriginals [20]. Callaghan’s sample contained a large proportion of Aboriginal clients who were referred to the service by other sources such as from physicians or social service or mental health care providers. However, in the present study, the majority of the Aboriginal people (96%; data not shown) were self-referred. In addition, our study focused exclusively on urban Aboriginals, whereas Callaghan’s data was from a rural setting and included 30% who reported their primary residence as “on-reserve”. Further study is warranted to clarify the different treatment needs of urban and rural Aboriginals as well as the impact of being “Status Indians” on reservations. For the non-Aboriginal group, our findings are in accord with other studies which found that younger patients [15,16] were more likely to leave AMA, and those with opiates as primary drug of choice were more likely to not complete their scheduled treatment [36].

The following limitations merit discussion. First, the information on substance use and drug-related diseases were based on self-report. However, previous studies have shown that patients’ self-reports of drug use are reasonably reliable and valid to provide descriptions of drug use, drug-related problems and the natural history of drug use [37]. Self-reported HIV and HCV status, however, likely under-represent the true prevalence of these infections [38]. Second, we did not have data on medication used during treatment and it is possible that this could influence treatment dropout [15]. Third, this study did not include assessment of psychiatric diagnoses, which previous literature has shown to be associated with both pretreatment and treatment dropouts [39,40].

## Conclusions

High pretreatment and treatment dropout rates may reflect the fact that the current addiction treatment system fails to retain the clients. Therefore, examining risk factors that are associated with pretreatment and treatment dropouts for Aboriginal and non-Aboriginal clients with substance use disorders have very important policy implications. It can help policy makers better understand why the system fails to retain the client and design and initiate culturally appropriate services to improve the current addiction treatment system. Specifically, our results highlight the importance of social factors, such as homelessness and timing of welfare check issuance for the Aboriginal population compared to substance-specific factors (drug of choice, for example) for the non-Aboriginal population. Although the study findings were drawn from a detox service in Vancouver DTES almost 10 years ago and since then social housing and addiction services in Vancouver have expanded [27]; however, factors associated with pretreatment and treatment dropouts, such as overrepresentation of the Aboriginals in Vancouver DTES, timing of welfare check issuance, and waiting times for detox still exist, and therefore our findings remain pertinent in providing information that can help clinicians and decision-makers design and initiate culturally appropriate services to minimize pretreatment and treatment dropout rates and therefore to improve the current addiction treatment system for both Aboriginal and non-Aboriginal clients, thereby improving clients’ physical and mental health outcomes and increasing cost-effectiveness of treatment resources.

## Competing interests

The authors declare that they have no competing interests.

## Authors’ contributions

XL took part in the conception of the study design and interpretation of results and drafted the manuscript. HS undertook statistical analyses and took part in conceiving the study design and interpreting results. DM interpreted results and was involved in the critical revision of the article for important intellectual content. AA was involved in the critical revision of the article for important intellectual content. All authors read and approved the final manuscript.
